# Endoplasmic Reticulum Stress-Unfolding Protein Response-Apoptosis Cascade Causes Chondrodysplasia in a *col2a1* p.Gly1170Ser Mutated Mouse Model

**DOI:** 10.1371/journal.pone.0086894

**Published:** 2014-01-27

**Authors:** Guoyan Liang, Chengjie Lian, Di Huang, Wenjie Gao, Anjing Liang, Yan Peng, Wei Ye, Zizhao Wu, Peiqiang Su, Dongsheng Huang

**Affiliations:** 1 Department of Orthopedics, Sun Yat-sen Memorial Hospital of Sun Yat-sen University, Guangzhou, Guangdong, China; 2 Department of Breast Surgery, Sun Yat-sen Memorial Hospital of Sun Yat-sen University, Guangzhou, Guangdong, China; 3 Department of Orthopedics, the First Affiliated Hospital of Sun Yat-sen University, Guangzhou, Guangdong, China; Duke University Medical Center, United States of America

## Abstract

The collagen type II alpha 1 (*COL2A1*) mutation causes severe skeletal malformations, but the pathogenic mechanisms of how this occurs are unclear. To understand how this may happen, a *col2a1* p.Gly1170Ser mutated mouse model was constructed and in homozygotes, the chondrodysplasia phenotype was observed. Misfolded procollagen was largely synthesized and retained in dilated endoplasmic reticulum and the endoplasmic reticulum stress (ERS)-unfolded protein response (UPR)-apoptosis cascade was activated. Apoptosis occurred prior to hypertrophy, prevented the formation of a hypertrophic zone, disrupted normal chondrogenic signaling pathways, and eventually caused chondrodysplasia. Heterozygotes had normal phenotypes and endoplasmic reticulum stress intensity was limited with no abnormal apoptosis detected. Our results suggest that earlier chondrocyte death was related to the ERS-UPR-apoptosis cascade and that this was the chief cause of chondrodysplaia. The *col2a1* p.Gly1170Ser mutated mouse model offered a novel connection between misfolded collagen and skeletal malformation. Further investigation of this mouse mutant model can help us understand mechanisms of type II collagenopathies.

## Introduction

To date, there are at least 339 records of collagen type II alpha 1(*COL2A1*) mutation types [1]. Such mutations alter the gene encoding the α1 chain of procollagen type II producing various chondrodysplasias, which can be lethal (hypochondrodysplasia) or deforming (spondyloepihyseal dysplasia congenital, and Kniest dysplasias, and Stickler syndrome) or simply as mild hip and knee joint diseases [2], [3], [4]. Although such phenotypes vary, a disordered growth plate and slowed endochondral ossification are commonly seen [5], [6], [7], [8]. Understanding chondrodysplasia requires clarifying the relationship between mutated collagen and the malformed growth plate.

To study the *COL2A1* mutation phenotype and the possible mechanisms behind it, at least 13 different mouse models have been reported (see Table S1) [5], [6], [7], [8], [9], [10], [11], [12], [13], [14], [15], [16], [17], [18], [19], [20], [21], [22], [23], [24], [25], [26], [27]. Most transgenic mice have a phenotype of chondrodysplasia/spondyloepiphyseal dysplasia, with nonfatal malformations in heterozygotes, and lethal deformities in homozygotes. In summary, the severity of the phenotype in these transgenic mice may involve several factors: the mutation type (large deletion > point mutation); mutation position (C-propeptide > Gly substitution > Y-position > X-position); and whether normal type II collagen exists (homozygotes > heterozygotes; homologous recombination/mutagenesis > traditional transgene). Interestingly, deformed growth plates with an abnormal cell ratio and disordered orientation of proliferative cells were common phenomena in these models, in spite of diverse severities. This general character of abnormal cell behavior caused by different mutations implies that the abnormal cell behavior may explain irregular skeletal development. Thus, more insight into *col2a1-*mutated chondrocytes is needed.

Mutated collagen II can affect cell behavior through a series of reactions that lead to apoptosis. Previously, researchers reported that mutated collagen excessively accumulated in dilated endoplasmic reticulum (ER) whereas collagen excreted into the extracellular matrix decreased sharply [8], [15], [20]. Further investigations indicated that the mutant molecules which retained in ER could induce ERS and activate a signaling network of the unfolded protein response (UPR) to eliminate misfolded collagen and maintain homeostasis [5], [26]. Once the mutated protein was continuously synthesized and homeostasis was perturbed, apoptosis was initiated. However, the influence of the ERS-UPR-apoptosis cascade in mutated chondrocytes is still uncertain, so more information is needed to clarify the relationship between mutated collagen induced apoptosis and chondrodysplasia.

We here describe a novel incomplete dominant inherited line of gene knock-in mice harboring the *col2a1*p.Gly1170Ser mutation. We provide comprehensive evidence of the ERS-UPR-apoptosis cascade in chondrocytes of homozygotes. When chondrocytes underwent apoptosis before hypertrophy, the hypertrophic zone disappeared in the growth plate. We suspected that untimely cell apoptosis prevented formation of a hypertrophic zone and disrupted normal chondrogenic signaling pathways. Therefore, the growth plate in the *col2a1* mutated mouse was abnormally developed.

## Materials and Methods

This series of studies was approved by the ethics committee of Sun Yat-Sen University, and all procedures involving animals met the relevant guidelines for humane care of laboratory animals.

### Construction of *col2a1* p.G1170S knock-in Mouse

A fragment containing the *col2a1* gene was first isolated from a bacterial artificial chromosome (BAC) cloned. Then, the p.G1170S missense mutation was introduced and the *col2a1*-Knock-in vector was constructed according to a recombination-based approach, as previously described [5], [28], [29]. Embryonic stem (ES) cells (SCR012, derived from the 129 Sv/Ev mouse strain) were transfected with the linearized targeting vector by electroporation (Bio-Rad Gene Pulser, 240 V/500 µF, 45 µg DNA per 2×10^7^ cells). A selection with G418 (300 µg/ml) and ganciclovir (2 µmol/L) was maintained for 8 days. Double-resistant colonies were selected, expanded, and analyzed for the presence of the recombination event by PCR mutant exon sequencing. Primers for 5′arm PCR verification: (Neo-R)5′-CTGAGCCCAGAAAGCGAAGGA-3′, (5L)5′-AGGGGGCGCCAGAGGGCAGTAAAG-3′; primers for 3′arm PCR verification: (Neo-F)5′-CCTCCCCCGTGCCTTCCTTGAC-3′, (3R)5′-CTGCGCCCAGCATCTGTAGGGGTCTT-3′; primer for sequencing: 5′-GGTCCACCTGGCCCTGTT-3′. Chimeric mice were generated by microinjection of homologous recombinant ES cells into C57BL/6J blastocysts, which were implanted into the uterine horn of pseudopregnant foster mothers. Chimeras were then mated with C57BL/6J wild-type females and germ-line transmission was confirmed by agouti coat color and genotyping. Genomic DNA was isolated from the mouse tail, and genotyping was performed by PCR product sequencing. Heterozygous offspring were interbred to generate homozygous mutated mice. Animals were housed in a temperature- and humidity- controlled room under a 12-h light-and-dark cycle with food and water *ad libitum*.

### Morphological Measurements

Fetuses, neonates and adult mice were euthanized, genotyped and measured with a Vernier caliper and an electronic scale. Data for height, the lengths of right femurs and humeri, and total animal weights were collected.

### Skeletal Analysis

Fetuses and euthanized neonates were genotyped, skinned, eviscerated and fixed in 95% EtOH for 3 days. Then, the mice were transferred to acetone and incubated overnight to remove fat. Alcian blue staining was performed in a solution of 80% EtOH, 20% acetic acid, and 0.015% alcian blue for 4 days. Specimens were rinsed and soaked in 95% EtOH for 3 days. Alizarin red staining was then performed overnight in a solution of 0.002% alizarin red and 1% KOH. After rinsing with water, specimens were kept in 1% KOH solution until the skeletons became clearly visible. Specimens were transferred into glycerol: ethanol (1∶1) for documentation and storage.

### Histological and Immunohistochemical (IHC) Analysis

Limbs were fixed in 4% paraformaldehyde for 24 h and decalcified in 10% EDTA for 3 days. Paraffin sections (4-µm) were obtained and stained with Hematoxylin and Eosin (H&E), safranin O and toluidine blue. Mean values of heights of the reserve, proliferative, hypertrophic zones, and the total growth plate were calculated from measurements taken at 3 positions across the proximal tibia growth plates using ImageJ version 1.44p software. IHC/Immunocytochemistry (ICC) was performed with Hsitostain-Plus kit (ZSGB-BIO, China). Primary antibodies included: collagen II (Sigma, USA), sox 9 (Abcam, UK), collagen X (Abcam, UK), and activated-caspase 3 (Bioworld, USA). Detection was conducted with a DAB horseradish peroxidase color development kit (ZSGB-BIO, China). Semi quantitative analysis of IHC images through integrated optical density (IOD) was taken using Image-Pro Plus version 6.0.0.260 software.

### Cell Culture

Chondrocytes isolated from embryos were cultured for five days with DMEM/10% FBS. Cells were then fixed and analyzed under confocal microscopy with anti-collagen II and anti-Grp78.

### TUNEL and EdU Labeling Assay

A TUNEL assay was performed according to the manufacturer’s instructions (MBL, Japan). An EdU labeling assay was performed with a Click-iT EdU Imaging Kit (Invitrogen, USA). Pregnant (19 day’s gestation) female mice were injected intraperitoneally (ip) with 100 µg/g EdU 3 h before being euthanized. Limb sections were manipulated according to the manufacturer’s instructions.

### Electron Microscopy

Electron microscopy analysis was performed, by standard procedures on growth plate cartilage from the lower limbs of newborn mice. Ultra-thin sections were stained with uranyl acetate and lead citrate, and examined using a Tecnai transmission electron microscope (FEI, USA) operated at 80 kV.

### Immunofluorescence and Confocal Microscopic Analysis

Chondrocytes were isolated from the articular cartilage of 3 genotypes, and cultured for 5 days with Dulbecco’s modified eagle medium (DMEM) containing 10% fetal bovine serum (FBS). Cells were labeled with mouse anti-collagen II (Sigma-Aldrich, USA) and rabbit anti-Grp78 (BIP, a chaperone serving as an ER marker; Epitomics, USA). Secondary antibodies were Alexa 594 for collagen II and Alexa 488 for Grp78 (Invitrogen, USA), respectively, followed by staining with DAPI. Signals were captured with a Zeiss LSM710 confocal laser scanning microscope.

### Real-time RT-PCR Assay

Total RNA was isolated from rib cartilage from neonates using RNAiso Plus reagent (TaKaRa, China) and converted to cDNA. Real-time PCR was performed on a Roche LightCycler 480 System using SYBR Green Real-time PCR Master Mix (TOYOBO, Japan).The expression of *Chop, Total-Xbp1, Spliced-Xbp1, Grp78 (BIP), ATF4* and *ATF6,* was detected to measure ERS. Expression of the *GAPDH* gene was used as a reference. Each reaction was processed in triplicate, and an average ΔCt value from the whole group was used. Relative gene expression was obtained for each using the 2^−ΔΔCt^ method.

### Statistical Analysis

Data distributions were expressed as the mean ± standard deviation of the mean (SD), and level of significance was set at *P*<0.05. Mean values of the groups were compared with two independent samples *t* test (for two groups) or one-way ANOVA, and subsequent pairwise mean comparisons performed by *post hoc* (Bonferroni) tests (for 3 groups) using SPSS for Windows statistical software package, version 13.0.

## Results

### Generation of Transgenic Mice

**Figure 1:**
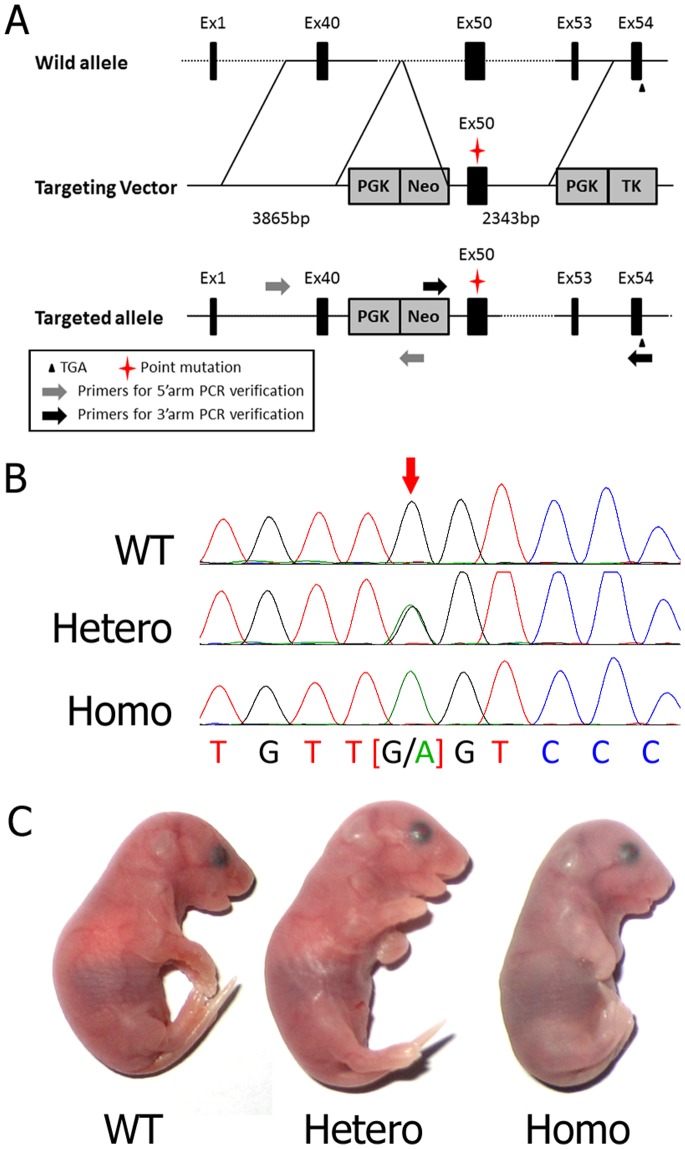
Construction of mutated mouse model. (A) Schematic description of transgene construction. A c.3508G>A mutation in Ex50 was generated by PCR-based site-directed mutagenesis and introduced into the targeting vector containing exons form Ex40 to Ex53 of the *col2a1* gene. A PGK-Neo gene and a PGK-TK gene were also introduced for the positive-negative selection. After being transfected into ES cells, the mutation was transferred into the genome by homologous recombination. The double resistant colonies which were verified with 5′arm+3′arm PCR and sequencing were considered correctly generated and further used for microinjection. (B) Sequencing results of mice of the 3 genotypes. Arrow denotes the c.3508G>A mutation in Ex50. (C) Phenotypes of the neonates of 3 genotypes. Homozygotes were cyanotic immediately after birth and died rapidly; heterozygotes had normal phenotypes.

The targeting construct represented 13.998 kb of mouse genomic DNA and contained exons (Ex) from Ex40 to Ex53 of the *col2a1* gene. Figure 1A depicts the targeting strategy: the homologous recombination between the targeted locus and the targeting construct leads to a modified gene that contained a positively selectable PGK-Neo gene. The p.G1170S missense mutation, encoded by a GGT → AGT change, was located in Ex50. The targeting construct contained a copy of the negatively selectable PGK-TK gene, allowing the use of a positive-negative selection of homologous recombinant ES clones. Ninety-six double-resistant ES clones were successfully amplified. The screening for the targeting event was conducted with PCR and sequencing, and 9 independent ES clones were validated. After blastocyst re-implantations, 11 viable chimeras were obtained and bred with wild-type mice (“WT”). Finally, heterozygous mice (“Hetero”) were characterized, and homozygous mutated mice (“Homo”) were generated by interbreeding heterozygous founders (Figure 1B). Litters from heterozygous offspring were normal in number. Neonates of heterozygotes survived and appeared normal, whereas homozygous offspring died shortly after birth from respiratory distress (Figure 1C). The dead homozygotes were severely dwarfed, with shortened trunks and limbs, hypoplastic thoraces, distended abdomens, cranial bulges, short snouts, truncated facial bones and cleft palates.

### Abnormal Physical Development and Skeletal Features of Mutant Fetuses

**Figure 2:**
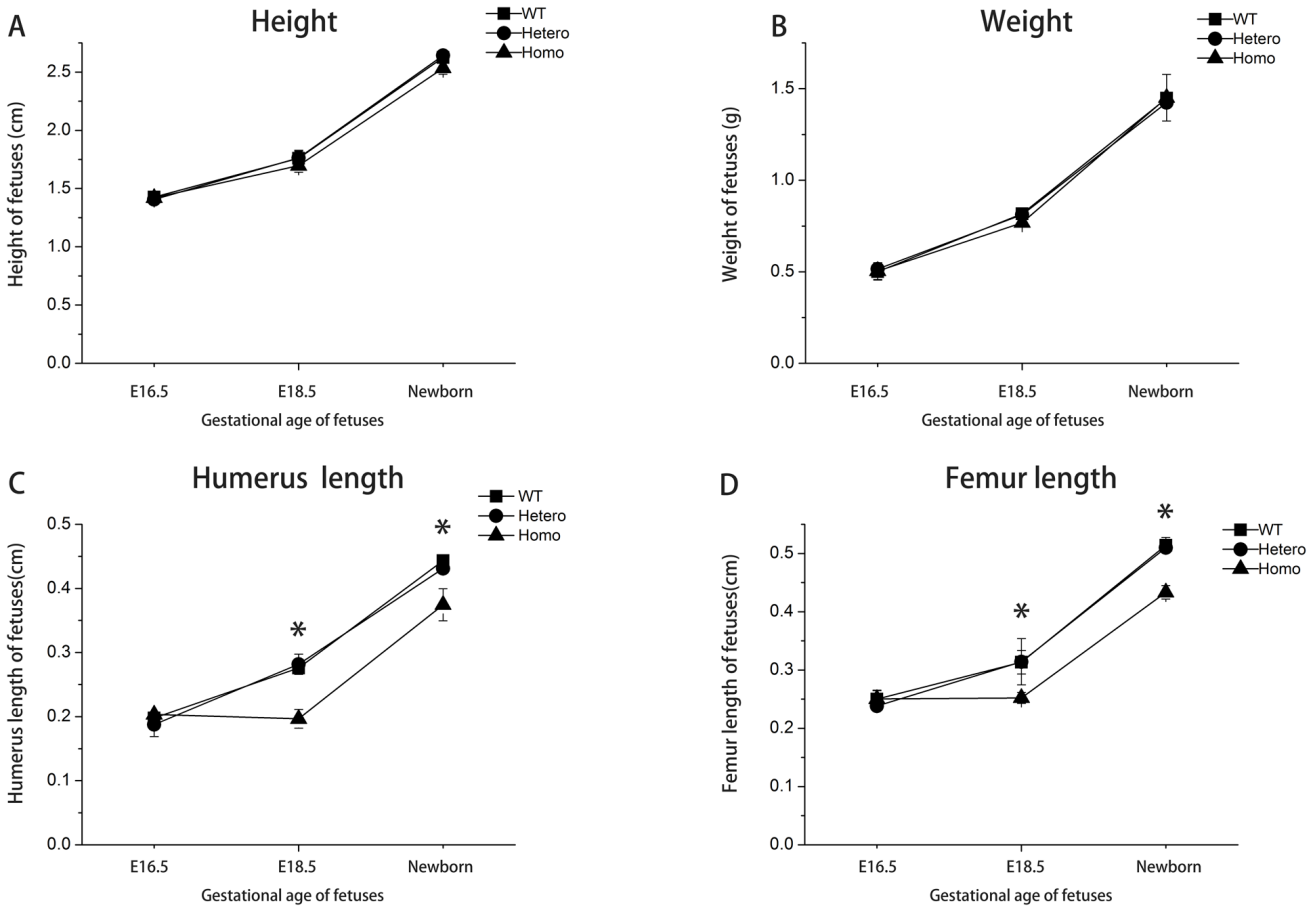
Morphological measurements of mutant fetuses and mice. (A) Heights of E16.5, E18.5 embryos and newborns. (B) Weights of E16.5, E18.5 embryos and newborns. (C) Humeri lengths of E16.5, E18.5 embryos and newborns. (D) Femur lengths of E16.5, E18.5 embryos and newborns. Sample size ≥3 littermates/genotype/time point. *P<0.05 was considered statistically significant.

Fetuses at E16.5, E18.5, and newborns were collected for morphological measurements. No obvious differences were observed with respect to height and weight at all time points among the 3 genotypes (Figure 2, A–B). Humeri and femurs lengths also did not differ significantly among the 3 genotypes at E16.5. However, the humeri and femurs were significantly shorter in homozygotes at E18.5 and in newborns (*P*<0.05; Figure 2, C–D). No significant difference was observed with respect to weight, height and long bone length of different genotypes of adult mice (data not shown). The shortening of the homozygous long bones suggested that the process of endochondral ossification was severely disturbed.

**Figure 3:**
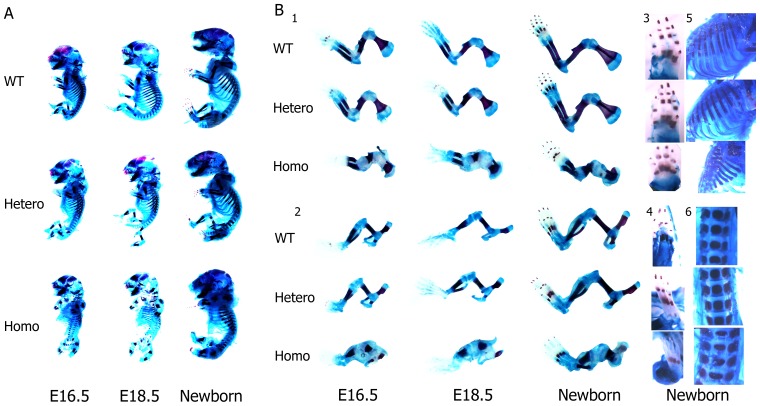
Skeletal analysis of mutant fetuses. (A) Alizarin Red and Alcian blue staining results of E16.5, E18.5 embryos and newborns. (B) Details of the skeletal structures between 3 genotypes: 1. forelimbs 2. hindlimbs 3. front paws 4. hind paws 5. ribs 6. lumbar spines. Note that in homozygotes, the middle phalanges were non-ossified, the intercostal spaces were decreased, and the vertebrae were shortened and widened. Heterozygotes were normal.

Fetuses at E16.5, and E18.5, and newborns were obtained for skeletal analysis. In homozygotes, alcian blue/alizarin red staining revealed severe defects in skeletal development (Figure 3, A–B). The differences were discernible since E16.5 and became more pronounced with growth. These fetuses were smaller, with shortened and widened long bones, abnormal scapulae, shapeless pelvises, non-ossified middle phalanges, malformed ribcages, and less mineralized vertebrae. These anomalies indicated that cartilage shaping was disturbed in mice lacking normal collagen II and endochondral ossification was slowed. As previously expected, intramembranous ossification was not affected. Heterozygotes were not abnormal and were difficult to distinguish from wild types by appearance.

### Abnormal Histological Structure of the Mutant Fetus with Disappearance of the Hypertrophic Zone

**Figure 4:**
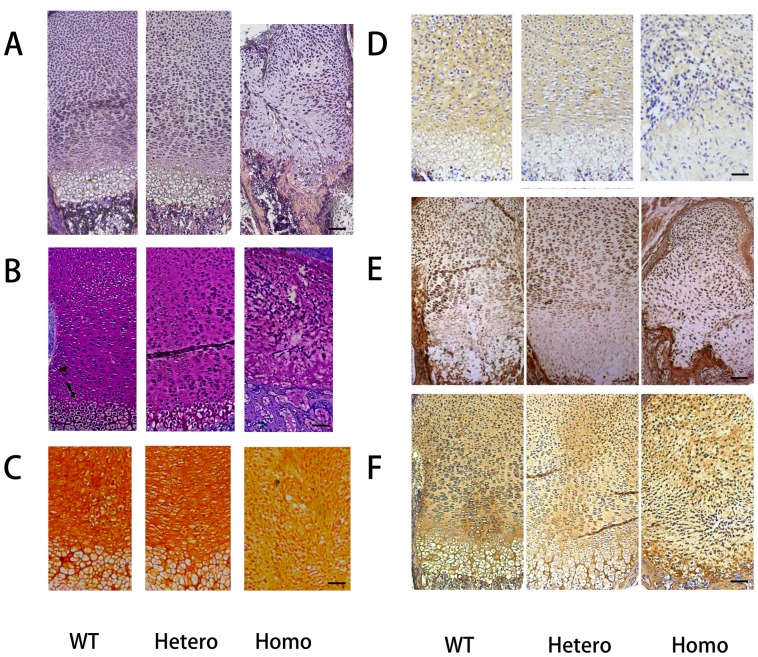
Histological study of growth plate. (A) H&E staining of the growth plates from E19.5 embryos. (B) Toluidine blue staining and (C) Safranin O staining of the growth plates showed decreased proteoglycans in mutated mice. (D–F) IHC analysis of the type II collagen (D), Sox9 (E), and type X collagen (F) were abnormally expressed in homozygotes. All scale bars = 100 µm.

**Figure 5:**
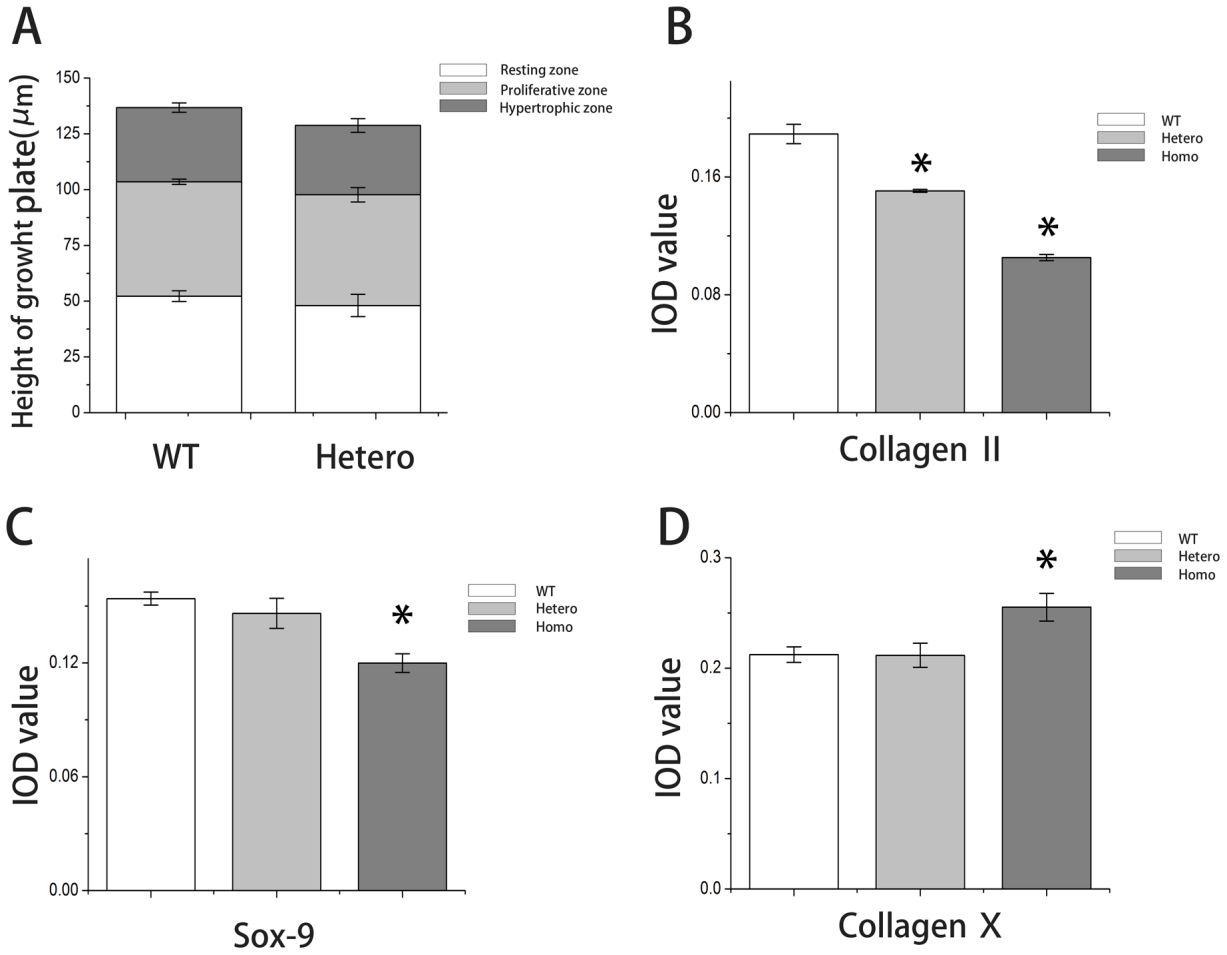
Quantitative analysis of growth plate and immunostaining. (A) Total growth plate and chondrocyte zone heights of wild types and heterozygotes. (B) Integrated optical density (IOD) values of collagen type II for all 3 groups. (C) IOD values of Sox9 for all 3 groups. (D) IOD values of collagen type X for all 3 groups. Sample size ≥3 littermates/genotype. IOD value of each sample was obtained from the average of 3 different sections under the same power lens. *P<0.05 was considered statistically significant.

Histological analyses of E19.5 mice revealed that the normal architecture disappeared in the homozygous growth plate (Figure 4A). Chondrocytes in the resting zone of the homozygotes seemed to be normally distributed. However, the proliferating chondrocytes became fusiform, decreased in number, and aligned transversely and chaotically. The hypertrophic zone was lost; although, several hypertrophic chondrocytes could be observed at the boundary between the cartilage and the ossification zone. Trabecular bones could not form properly because of loss of regular alignment of hypertrophic cells. However, there was no remarkable change in heterozygotes, and the heights of each zone remained similar to wild types (Figure 5A). Toluidine blue and safranin O staining revealed that proteoglycans were reduced severely in homozygotes, but heterozygotes appeared similar to wild types (Figure 4, B–C). Type II collagen decreased in both heterozygotes and homozygotes (IOD values reduced ∼ 18.4 and 43.0%, respectively, when compared with wild types) (Figure 4D&5B). Expression of Sox9 (which regulates the expression of type II collagen) in the growth plate was significantly decreased in homozygotes (Figure 4E&5C). Although hypertrophic cells were barely generated in homozygotes, they expressed almost 20% more type X collagen in the hypertrophic zone (Figure 4F & 5D).

**Figure 6:**
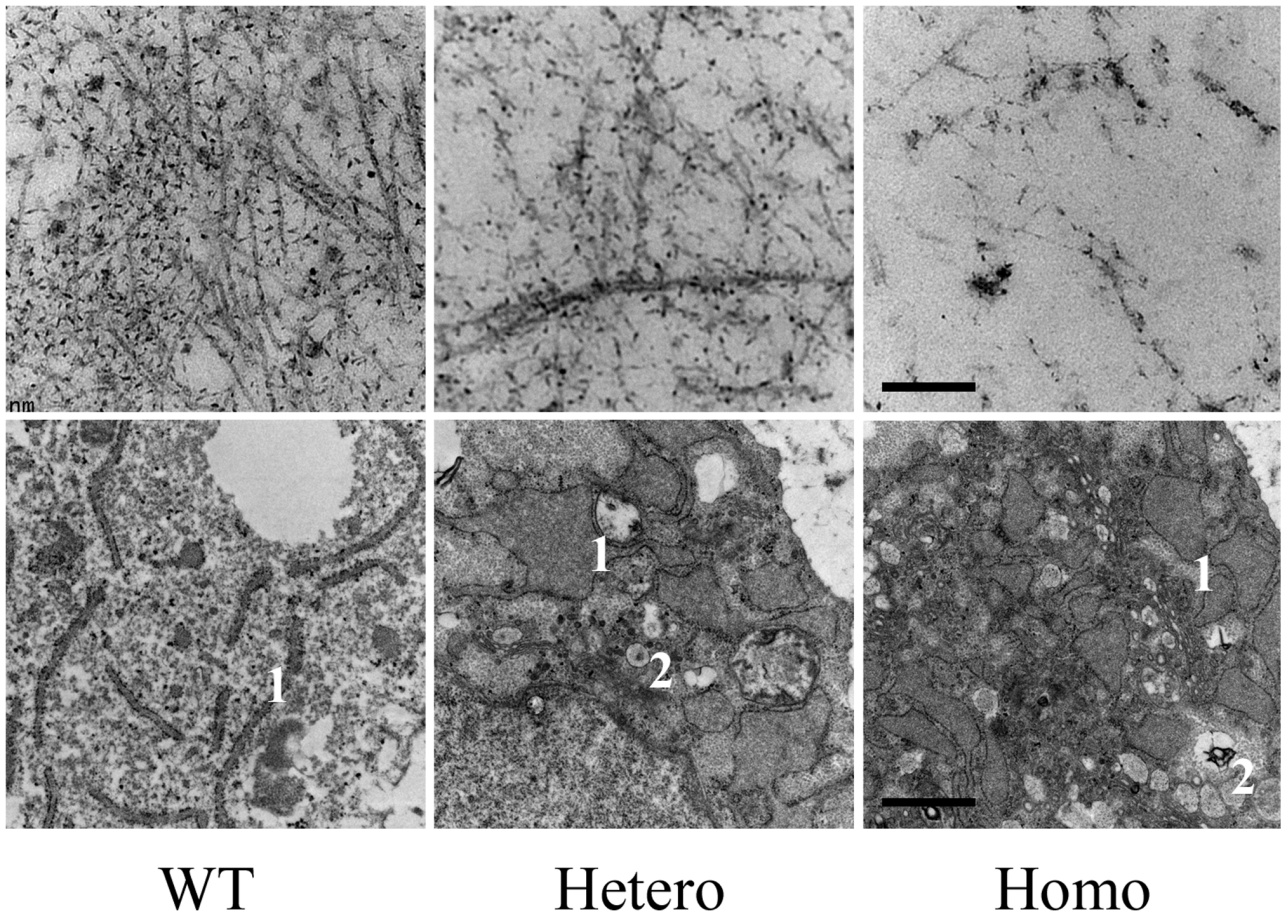
Transmission electron microscope analysis. Transmission electron microscope analysis of the extracellular matrix (A, B, C) and chondrocytes (D, E, F) in the proliferating zone of the growth plates from E19.5 embryos. Dilated vesicles, such as the ER (1), and Golgi body (2), were commonly seen in transgenic chondrocytes (E, F). Scale bar for a–c corresponded to 1 nm, and bar for D–F corresponded to 200 nm.

**Figure 7:**
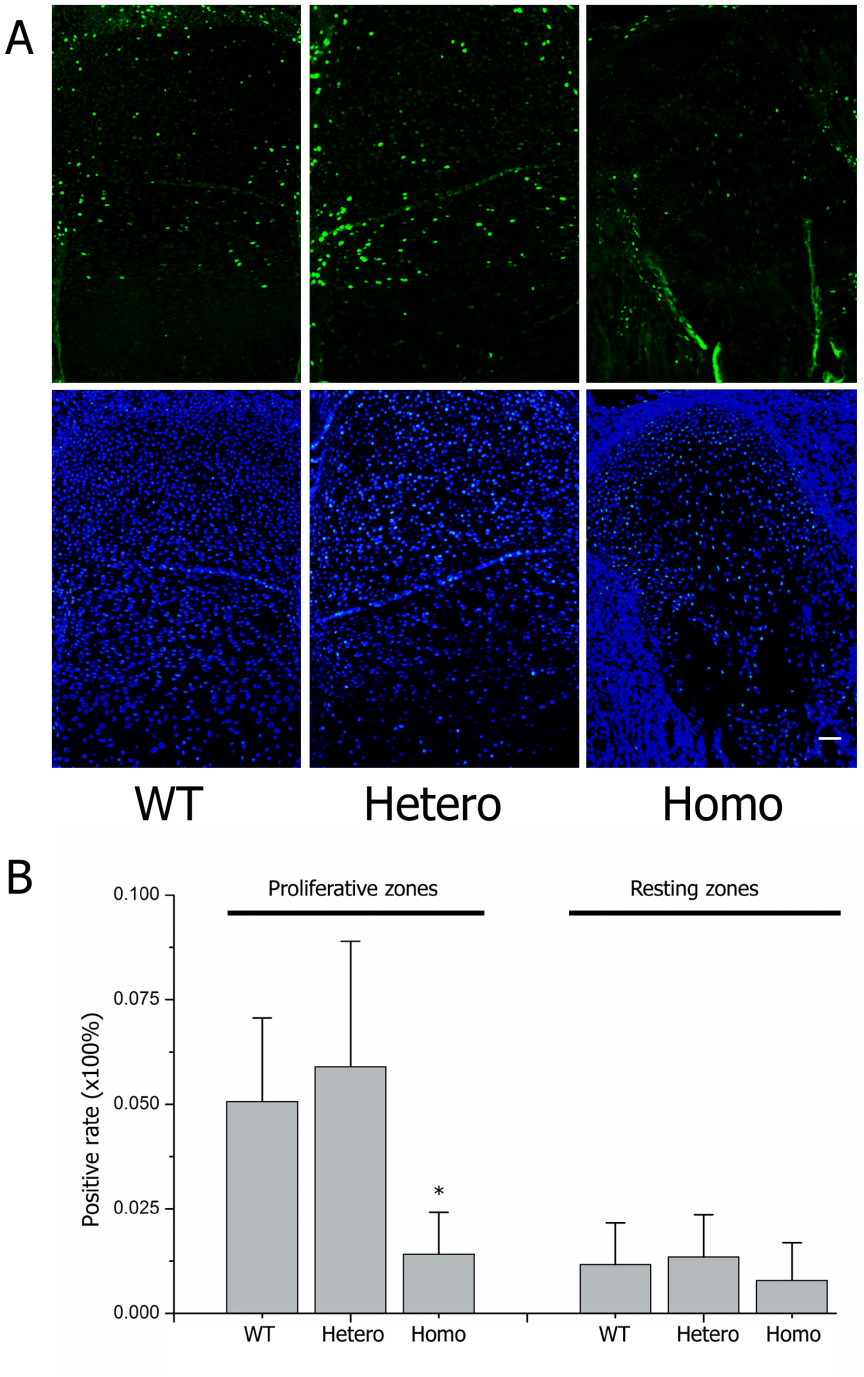
EdU study in growth plates. Short term labeled (3 h) EdU assay results in growth plates of E18.5 embryos. (A) Green fluorescent signals indicated proliferated cells and DAPI staining for nucleuses. (B) Statistical analysis of the positive rates within littermates (nWT = 2/nHetero = 5/nHomo = 3, each limbs had ≥3 sections for analysis) showed that proliferating chondrocytes were significantly decreased in homozygotes (*P<0.01).

Transmission electron microscope analysis depicted fewer collagen fibers and proteoglycan aggregates in mutated cartilage, and abnormal type II collagen in homozygotes was assembled into aberrant bundles (Figure 6, A–C). An EdU assay showed that the rate of cell multiplication in the homozygous proliferating zone decreased significantly, indicating fewer proliferating chondrocytes in the homozygous growth plates (Figure 7, A–B). These data show that collagen II expressing cells, i.e., proliferative cells, decreased in the mutated cartilage before they could differentiate into hypertrophic cells.

### ERS-UPR-apoptosis Cascade in Mutated Chondrocytes

**Figure 8:**
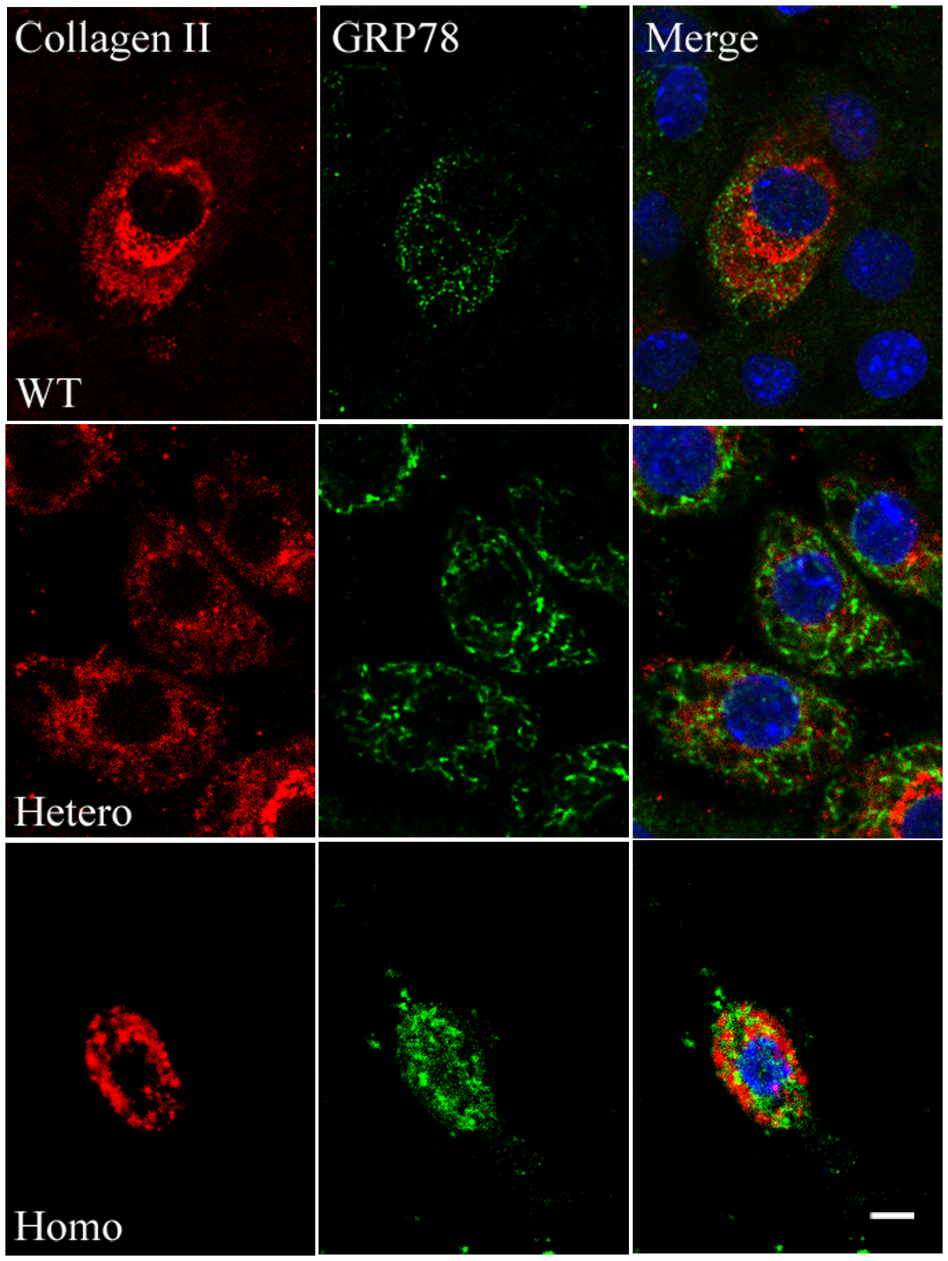
Confocal microscope analysis in chondrocytes. Confocal microscope analysis results of chondrocytes that were isolated from articular cartilages of E19.5 embryos, cultured for 5 days, and processed with immunofluorescence with antibodies for type II collagen (left) and Grp78 (middle, an ER marker). Merged photos (right) showed abnormal assembly and intracellular retention of mutated type II collagen in homozygous cells. Scale bar = 10 µm.

**Figure 9:**
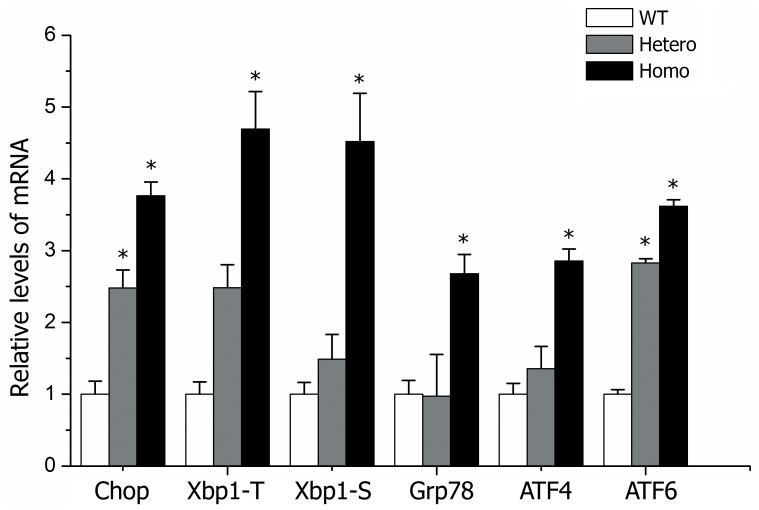
Relative ERS-related genes in rib cartilages of littermates. Each genotype contained more than 3 littermates. Gene expression was measured by real-time quantitative RT-PCR and normalized to GAPDH expression. Relative expression was calculated using the 2-ΔΔCt method. *P<0.05 was considered statistically significant.

**Figure 10:**
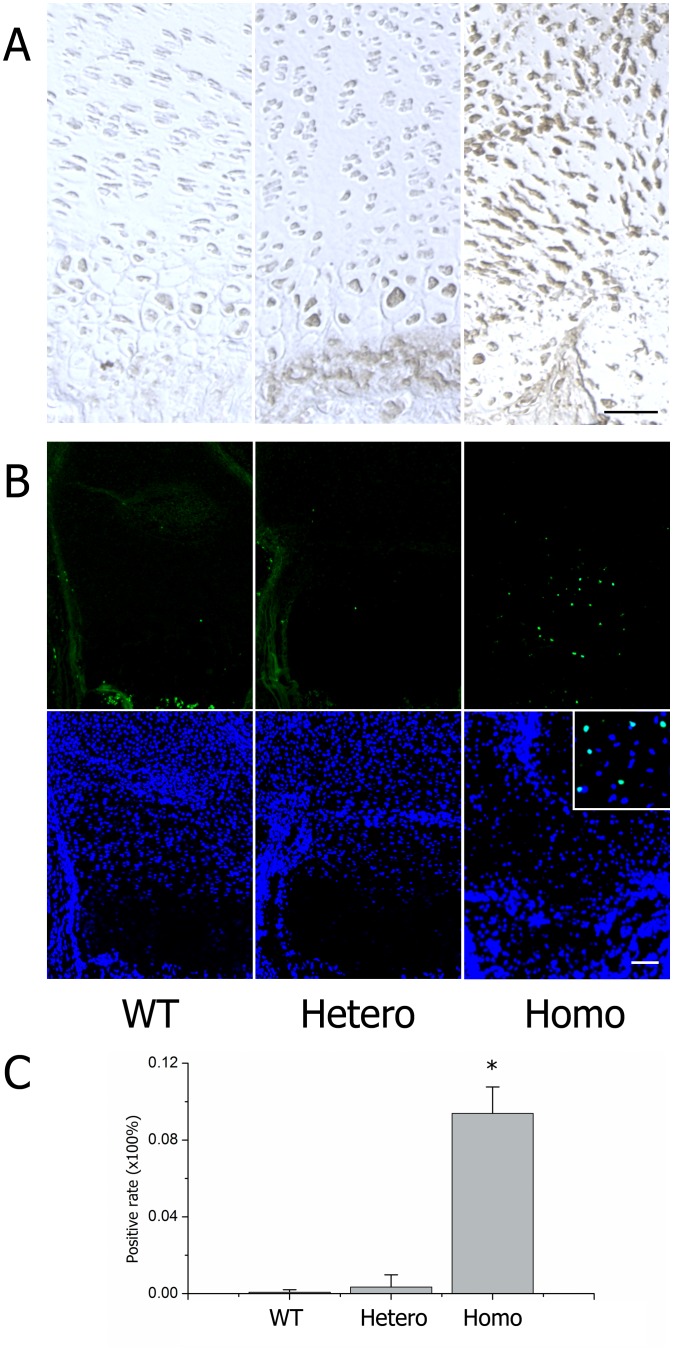
Experimental evidence for apoptosis. (A) Immunostaining of cleaved caspase-3 in the growth plates. (B) TUNEL assay results showed apoptosis chondrocytes (green fluorescence) with DAPI labeled nucleuses. (C) Statistical analysis of the positive rates within littermates (≥10 sections for each genotype) showed increased apoptosis in homozygotes (*P<0.01). Scale bar = 100 µm.

To ascertain why proliferative cells decreased and the hypertrophic zone disappeared, we examined programmed cell death. Previously, investigators reported that mutated collagen was retained in the ER and caused ERS, leading to an UPR signaling network, causing apoptosis [26], [27]. In our mouse model, we observed that the rough ER in mutated chondrocytes was dilated and glycogen granules abnormally accumulated, especially in homozygotes (Figure 6, D–F). Confocal microscope analysis of cultured chondrocytes collected from embryonic cartilage showed that the mutated type II procollagen in homozygotes assembled into bundles, co-localized with the ER, and was retained intracellularly (Figure 8). Expression of several ERS-related genes, including *Chop, Total-Xbp1, Spliced-Xbp1, Grp78 (BIP), ATF4, ATF6,* were all up-regulated in homozygous cartilage and partly increased in heterozygotes (Figure 9). Cleaved caspase-3 was increased in homozygous cartilage, indicating that the caspase cascade was activated (Figure 10A). An increase in apoptotic cells in the homozygous growth plate was confirmed with TUNEL assay (Figure 10, B–C). Nevertheless, activated caspase-3 and apoptosis were similar in heterozygotes and wild types. These results indicated that in homozygotes, the ERS-UPR-apoptosis cascade reduced proliferative chondrocytes before they could differentiate into hypertrophic chondrocytes. Thus, the hypertrophic zone disappeared and further deformed the growth plate. In heterozygotes which synthetized less mutant collagen, mild effects were seen. The ERS-UPR was activated as well, and cells could remain in homeostatic balance, avoiding apoptosis and malformation.

## Discussion

Here we report studies in a *col2a1* p.Gly1170Ser knock-in mouse model that we constructed to reveal possible mechanisms of how the *col2a1* mutation caused chondrodysplasia. Mutated procollagens were restrained in the ER, and subsequently ERS and UPR was activated to degrade misfolded proteins and keep cellular homeostasis. In homozygotes, the stress was severe enough to trigger apoptosis and proliferative chondrocytes underwent programmed cell death before they further transformed into hypertrophic cells. Eventually, disordered growth plates and chondrodysplasia occurred. In contrast, in heterozygotes, apoptosis was avoided after limited ERS, and the normal growth plate structure and endochondral ossification process were maintained.

Mutations of membrane and secretory proteins which synthesized in the ER can induce the ERS-apoptosis cascade which is thought to be important to diabetes, Alzheimer’s disease, osteogenesis imperfecta and others [30], [31], [32]. Mutation of extracellular matrix (ECM) genes (*col1a1*, *col2a1* and *col10a1*) has been shown to activate the ERS-apoptosis cascade, an important cause for ECM dysfunction [27], [30], [33]. Our work suggests that the ERS-apoptosis cascade may mediate the *col2a1* mutation to cause chondrodysplasia. In our mouse model, ERS was observed in homozygotes and heterozygotes, but the intensities were uniquely manifested in electron microscope graphs, confocal images, and with respect to elevated ERS-related genes. This difference, which may be derived from the different synthetic quality of mutated collagens, may explain the differences in apoptosis. Moreover, when compared with other *col2a1* mutations, though apoptosis would not always happen, ERS could be observed in almost all mutant models [20], [26], [27]. Taken these together, we concluded that mutation of *col2a1* which produced misfolded proteins caused ERS, but whether apoptosis occurred is more complex and may be associated with different mutation types and positions.

In our homozygous growth plate, apoptosis was the major cause for chondrodysplasia as it influenced the formation of proliferative and hypertrophic zones, and these data have been supported in other literatures [26], [27], [34]. However, deficiency of normal collagens in the cartilage matrix was also considered to cause disordered growth plates and chondrodysplasia in previous research [5], [6], [8], [20]. Of course, this may be an important reason, and we also observed an absence of normal collagen in the extracellular matrix. But, actually, when we reviewed their experimental results,abundant evidences for the existence of ERS, especially retained mutant collagens and dilated ER in electron microscopic images, could be observed widely [8], [12], [20]. Thus, ERS associated apoptosis may be at least another important pathway leading to chondrodysplasia. Almost all *col2a1* mutant homozygotes had similarly severe malformations such as dwarfism, short limbs, impaired endochondral ossification and even lethal deformities [5], [8], [15], [27]. For these cases, lack of normal collagens and extracellular structure appeared to be more important than apoptosis for skeleton malformation; a finding that was confirmed by Esapa who reported that *col2a1* Ser1386Pro mutant homozygotes developed typical chondrodysplasia without ERS associated apoptosis [5]. Heterozygous phenotypes varied considerably and this could not be explained by a deficiency of normal collagens. Other researchers have attributed this to dominant-negative effects of mutant collagen, but until now, evidence for this has been scarce [8], [20], [33]. Some reports suggest that in heterozygotes, mutant collagens in the cartilage matrix were less than 50% and the retention of misfolded proteins in enlarged ERs was observed [35], . It means that a substantial proportion of mutant collagen could not be secreted. Subsequently, degradation of retained proteins (the UPR) can induce ERS. Stress intensities were different due to variations in retained collagen arising from different kinds of mutations [37]. Thus, stress severity decided the occurrence of apoptosis. Additionally, thermostability of mutant procollagen may also influence induction of the ERS-apoptosis cascade [26], [34]. Moreover, some researchers have suggested that certain kinds of mutant collagens can incorporate with normal ones and secrete into the ECM, permitting heterozygotes to escape apoptosis [37], [38]. In summary, we speculate that ERS-associated apoptosis may offer a better explanation for the varied pathogenesis of heterozygotes.

Our study still has some limitations. For instance, we could not find or induce hip joint lesions of the human *COL2A1* p.Gly1170Ser mutation in this mouse model until now. However, we are investigating this different phenotype in future work. What’s more, collagen type II is not the only structural material for the cartilage matrix; it can modulate chondrogenesis and osteogenesis as an extracellular signal molecule [39], [40], [41]. Therefore, alterations of signaling pathways regulating endochondral ossification in the growth plate caused by the ERS-UPR-apoptosis cascade and/or a lack of normal collagen may be a promising research direction.

## Supporting Information

Table S1 **Summary of all the** ***COL2A1*** **mutated mouse model.**
(DOCX)
