# Supplementary material for: Endoplasmic Reticulum Stress-Unfolding Protein Response-Apoptosis Cascade Causes Chondrodysplasia in a col2a1 p.Gly1170Ser Mutated Mouse Model
Source: PLoS One. 2014 Jan 27;9(1):e86894. doi: 10.1371/journal.pone.0086894 (PMC3903611; doi:10.1371/journal.pone.0086894)
Supplement: Table S1 — Summary of all the COL2A1 mutated mouse model. (DOCX) [file pone.0086894.s001.docx]

**Table S1. Summary of all the *COL2A1* mutated mouse model**

| Number | First published year | Author | Mouse | Generation method | cDNA^a^ | Protein^b^ | Mutation effect | Heterozygotes phenotype^c^ | Homozygotes phenotype |
| --- | --- | --- | --- | --- | --- | --- | --- | --- | --- |
| 1 | 1981 | Brown et al. | Disproportionate micromelia mice | Irradiation on spermatogonia | c.4341_4343del | p.Lys1447Thr1448Asn | Substitution | + | ++ |
| 2 | 1991 | Vandenberg et al. | Expressing partially deleted murine Col2a1 | Transgene by cosmid | intron15_intron27del | large (12 exons) deletion | Deletion | ++ | |
| 3 | 1991 | Garofalo et al. | Expressing missense mutated murine Col2a1 | Transgene by cosmid | c.853G>T | p.Gly285Cys | Gly Substitution | + | ++ |
| 4 | 1992 | Metsaranta et al. | Expressing partially deleted murine Col2a1 | Transgene by cosmid | c.610_654del | 15 amino acids (exon7) deletion | Deletion | + | |
| 5 | 1995 | Li et al. | Col2a1 knockout mice | Transgene with homologous recombination | A neomycin-resistance gene was inserted into exon35. | RNA transcripts terminated in exon35 | The pro-α1(II)chain cannot be generated. | + | ++ |
| 6 | 1997 | Maddox et al. | Expressing missense mutated murine Col2a1 | Transgene by cosmid | c.2320G>A | p.Gly774Ser | Gly Substitution | + | |
| 7 | 2002 | Gaiser et al. | Expressing missense mutated murine Col2a1 | Transgene by cosmid | c.2965C>T | p.Arg989Cys | Y-position Substitution | ++ | |
| 8 | 2002 | Arita et al. | Expressing mutated human Col2a1 | Transgene by cosmid | c.2155C>T (*Human*) | p.Arg719Cys (*Human*) | Y-position Substitution | - | ++ |
| 9 | 2003 | Donahue et al. | Spondyloepiphyseal dysplasia congenita mice | Naturally occurring | c.3574C>T | p.Arg1192Cys | X-position Substitution | - | + |
| 10 | 2003 | Barbieri et al. | Expressing partially deleted murine Col2a1 | Transgene by cosmid | intron49_intron50del | 36 amino acids (exon50) deletion | Deletion | ++ | |
| 11 | 2004 | Sahlman et al. | Replacing murine Col2a1 with mutated human Col2a1 | mating Arita's mice with Li's | c.2155C>T (*Human*) & mouse Col2a1 knockout | p.Arg719Cys (*Human*) | Y-position Substitution (Human) & knockout (mouse) | M^-/-^HMut^+/-^: ++;  M^+/-^HMut^+/-^: +;  M^-/-^HMut^+/+^: +; | |
| 12 | 2011 | Tatsuya Furuichi et al. | Point mutated at Col2a1 | ENU mutagenesis | c.4406A>C | p.Asp1469Ala | C-propeptide substitution | - | ++ |
| 13 | 2012 | Esapa et al. | Longpockets mice | ENU mutagenesis | c.4156T>C | p.Ser1386Pro | C-propeptide substitution | + | ++ |
| 14 | Our model | | Point mutated at Col2a1 | Transgene with homologous recombination | c.3508G>A | p.Gly1170Ser | Gly Substitution | - | ++ |

1. cDNA changes were renumbered from the first base of the start codon (ATG) according to the reference sequence of collagen alpha-1(II) chain isoform 1 precursor, i.e. NM_031163 (mouse) or NM_001844 (human).
2. Amino-acid changes were renumbered from the first Methionine of the reference sequence of collagen alpha-1(II) chain isoform 1 precursor, i.e. NP_112440 (mouse) or NP_001835 (human).
3. Description of the phenotypes of transgenic mice was simplified as: (-) not affected or have subtle problems uneasy to recognize; (+) viable, but with evident chondrodysplasia; (++) died at birth, with severe osteochondrodysplasia.
